# What is the perception of treatment in some European mental health services? The point of view of users belonging to ethnic minorities

**DOI:** 10.3389/fpsyg.2026.1664913

**Published:** 2026-03-31

**Authors:** Antonio Iudici, Giulia Gusella

**Affiliations:** 1 Department of Philosophy, Sociology, Education and Applied Psychology (FISPPA), University of Padua, Padua, Italy; 2 Institute of Psychology and Psychotherapy, Padua, Italy

**Keywords:** discrimination, ethnopsychology, inequalities, mental health services, user’s perspective

## Abstract

One of the most important criticism about mental health services regards the difficult that people meet when looking for a psychological support from these services. These health problems concern every citizen, but even more so for people with an ethnic background who are more exposed to discrimination, stigma and marginalization than the majority population of a country. For this reason, we decided to conduct research on scientific material on this topic, focusing on articles that shed light on the perspective of users belonging to ethnic minorities. The selected articles refer to some countries belonging to the European Union. Furthermore, the research focused on the main barriers that users reported perceiving when accessing mental health services. This paper offers a review of Europe literature concerning the need to estimate the main critical issues for which ethnic or migrant people are not receiving an appropriate help from mental health services. Another important characteristic is that the work aims to give a voice to the people involved in this review, which is why only articles and works that noted the user’s own perspective were considered. Our work found that people with different cultural background face specific barriers when seeking help from mental health services, in particular, we noted two main barriers that will be specifically explained. The results show that people with ethnic or migrant background have to face more and specific critical aspects when seeking psychological help compared to the main population of a nation. In particular, the criticism of being discriminated includes the criticism of being treated differently, unequally or disparately compared to the majority of population of a country. Even today, ethnicity plays a significant role in determining how people receive mental health care. The perception of receiving unfair and often ethnocentric treatment and the underrepresentation of ethnic minority patients in clinical services indicate that ethnic minorities face significant unmet healthcare needs, which may further contribute to the socioeconomic difficulties that some minority groups already face.

## 1 Introduction

Migration flows are the movement of people from one geographical area to another, which may occur within the same country (internal migration) or between different nations (international migration) (Czaika and De Haas, 2014). These migratory flows can be motivated by various factors, such as economic factors, whereby people move in search of better job opportunities, better economic conditions or greater economic stability (Dustmann and Görlach, 2016); social factors, involving the desire to reunite with family or to join communities with cultural affinities (Boccagni and Baldassar, 2015); political factors, which involve people leaving their country due to political persecution, discrimination or human rights violations (Crawley and Skleparis, 2018); and environmental factors, which involve migration caused by natural disasters, climate change or environmental degradation that make living in certain areas unfeasible (Castles and Miller, 2009; Cattaneo et al., 2019). Massey et al. (1998) explore the factors that determine the phenomenon of migration flow. Push and pull factors are key concepts in the study of migration and describe the forces that encourage people to leave their country of origin (push) and those that attract them to a new country of destination (pull) (Van Hear et al., 2018; Carling and Collins, 2018).

Push factors are unfavorable elements or difficult conditions in countries of origin that push people to leave their homeland (Castles et al., 2014). Some of the main ones are economic instability and poverty, such as unemployment and low wages, as a lack of job opportunities and low wages drive many individuals, especially young people, to seek better economic prospects abroad (Docquier et al., 2014); or famine and scarce resources, as in regions affected by a lack of resources or infrastructure, such as drinking water and sanitation, the quality of life is drastically reduced, which can push people to emigrate (Adger et al., 2015). In addition, conflicts and persecution, such as wars and violence, can be encountered. Civil wars, ethnic conflicts and armed uprisings force many people to leave their countries for safety (Adhikari, 2013).

When we talk about ethnic minorities, we mean a non-dominant group which is usually numerically less than the majority population of a State or region regarding their ethnic, religious or linguistic characteristics and who (if only implicitly) maintain solidarity with their own culture, traditions, religion or language (European Migration Network, 2019). Furthermore, ethnicity is a socially constructed concept and it is not biologically determined, but rather the result of historical, social and cultural processes. It is fluid and subject to change over time, it is often based on the individual’s self-identification, it can vary greatly within the same population. By the word “ethnic,” we mean the cultural group to which one belongs, and not the reification of belonging (Eriksen, 2010; Jenkins, 2008; Wimmer, 2013). Political, religious or ethnic persecution sees people belonging to persecuted political, religious or ethnic groups often forced to flee in order to avoid stigma, discrimination, arrests or even violence (Schmeidl, 2019). We use stigma concept as a mark of shame, disgrace or disapproval that results in an individual being rejected, discriminated against and excluded from participating in a number of different areas of society (World Health Organization/Europe, 2008). At the same time, for discrimination we refer to unfavorable or unjust treatment of a person or group based on protected characteristics such as race, color, nationality, sex, sexual orientation, age, disability, religion, or other categories specified by law (European Union, 2000; United Nations, 1965). In institutional terms, discrimination occurs when an individual or group is treated less favorably than others in comparable situations, without an objective and reasonable justification (International Labour Organization, 1958). In this research we use also the discrimination perception, that refers to an individual’s or group’s subjective awareness or belief that they have been treated unfairly or unfavorably due to their membership in a specific social group (Pascoe and Smart Richman, 2009).

These factors also include environmental degradation and natural disasters (Cattaneo et al., 2019). Pull factors, on the other hand, represent the favorable conditions in destination countries that attract people to emigrate (Carling and Collins, 2018).

Some of the main ones are economic and labor opportunities: countries with strong and diversified economies offer more employment opportunities, often with higher wages and better working conditions, and the presence of stable markets and opportunities for professional advancement exerts a strong attraction on migrants (Borjas, 2014; Kerr et al., 2017). In addition, nations with stable governments, low crime and respect for human rights are desirable places for those fleeing dangerous conditions and the guarantee of rights and justice attracts migrants seeking a pro-life and dignity (Ramos, 2018). Quality of life and social services are a significant pull factor, as the presence of quality schools and universities, efficient hospitals and accessible healthcare systems attract people eager to improve their lives and those of their families (Beine et al., 2014; Tani, 2017). With access to care, we mean the opportunity to identify health care needs, to seek health care services, to reach, to obtain or use health care services, and to have these needs fulfilled (Levesque et al., 2013). The interaction between push and pull factors plays a key role since these factors do not act in isolation, but are often interconnected (Van Hear et al., 2018). Migration flows can have economic, social and cultural impacts on countries of origin and destination, influencing aspects such as the labor market, demographics and local culture (Dustmann et al., 2016; Vertovec, 2019).

Ambrosini (2017) investigates in depth the social, economic and political implications of this phenomenon. In particular, he analyses the concept of integration and inclusion: he highlights how irregular migrants contribute to the host society in many ways, despite the fact that their position is often invisible or marginal. Many work in sectors such as domestic care and services, becoming an integral part of the informal welfare system (Ambrosini, 2013; Lutz, 2011). On the other hand, community relations see irregular migrants, who often face obstacles to inclusion, create networks of solidarity within migrant communities and sometimes even with the local population, giving rise to forms of mutual support (Ryan et al., 2008; Bloch et al., 2014). On the economic front, they make an informal economic and labor contribution; indeed, the presence of irregular migrants is significant in economic sectors such as agriculture, construction and personal care (Reyneri, 2004; Triandafyllidou, 2010).

Today, globalization and digitization have led to a view of culture as constantly fluid, hybrid and plural. Culture is now understood as a network of dynamic and interconnected meanings, which are transformed through migration, multiculturalism and interaction between different cultures (Baldwin et al., 2006; Spencer-Oatey and Franklin, 2012; Matsumoto and Juang, 2017). Ethnic minorities may have a strong and unique identity, and often maintain their cultural practices despite the pressure to assimilate into the dominant culture. However, these communities may face challenges related to discrimination, marginalization and unequal access to rights and resources.

The mental health of ethnic minorities in Europe is a complex issue, influenced by a range of social, economic and cultural factors. People from ethnic minorities often face additional challenges that may negatively affect their psychological well-being (Bhugra et al., 2011).

With regard to mental health care, minority populations face many difficulties in accessing services, including linguistic, cultural and economic barriers (Priebe et al., 2012). To address these challenges, there is a growing awareness of the need for culturally competent mental health services (Bhui et al., 2015). These services aim to understand and respect the specific experiences and perspectives of different communities.

The European Union has recognized the importance of this issue by launching various projects to improve mental health care for minorities. A significant example is the PROMO project (Best Practice In Promoting Mental Health In Socially Marginalized People In Europe), which sought to identify and promote best practices in this field (Priebe et al., 2013).

Finally, it is important to note that refugees and asylum seekers are a particularly vulnerable group, often requiring specific support for trauma and post-traumatic stress (Fazel et al., 2005).

Some of the main aspects linking mental health and ethnic minorities in Europe include, first of all, significant discrimination, which has a direct impact on their mental health. Structural racism and everyday incidents of discrimination can cause chronic stress, anxiety, depression and post-traumatic stress disorder (Williams and Mohammed, 2009). Continued exposure to these forms of oppression can lead to feelings of exclusion, alienation and social isolation. In addition, we can find other factors that result in barriers to accessing mental health services, such as language, cultural, economic and migration experience barriers (Marmot et al., 2010). In order to better understand the relationship between discrimination and the perception of discrimination, and to fully grasp the participants’ point of view, we used the interactionist perspective as a framework. The interactionist perspective offers a powerful framework for social research (Blumer, 1969; Mead, 1934; Iudici, 2015; Iudici and Fabbri, 2017; Iudici et al., 2020b). This perspective focuses on how individuals create and negotiate meanings through social interactions. According to symbolic interactionism, social reality is constructed through the interpretation and negotiation of shared symbols and meanings (Goffman, 1959; Berger and Luckmann, 1966).

In a research context, the interactionist approach emphasizes the importance of understanding individuals’ subjective experiences and how these are shaped by social interactions (Denzin, 2001). Researchers adopting this framework focus on observing and interpreting interaction processes, paying particular attention to the language, text, gestures, and symbols used by participants (Charmaz, 2014; Iudici and Gagliardo Corsi, 2017).

Methodologically, interactionism favors qualitative approaches such as ethnography, in-depth interviews, and discourse and text analysis (Atkinson and Housley, 2003; Iudici et al., 2020a). A key aspect of this approach is the recognition of the participants, but also researcher’s, active role in the research process (Holstein and Gubrium, 2008; Snow, 2001; Strauss, 1993).

The main objective is to highlight how the treatment in Mental Health Services is configured from the perspective of users belonging to ethnic minorities. Our interest is to highlight how users perceive the clinical intervention implemented by the services.

## 2 Methods

### 2.1 Research method: the literature review

According to the typology shared by Grant and Booth (2009) and the Joanna Briggs Institute line guides (Aromataris et al., 2024; Pearson et al., 2024; Joanna Briggs Institute, 2014), our work is to be considered as a literature review, which focuses on specific problematic issues ascribed to the theoretical and methodological complexities of the construct of Ethnology. According to the Medical Subject Headings scope note, a literature review describes published materials that provide an examination of recent or current literature (Lipscomb, 2000). In this case, our research can be considered a narrative review of textual evidence that is focused on the user’s perspective, which is not often reached by studies. Consequently, the synthesis used in this work is narrative, and the analysis carried out is of a conceptual nature (Hall and Walton, 2004). The aim is to systematize and summarize the available data, allowing new research to fill gaps and omissions.

### 2.2 Search strategy, criteria, and data collection

The review of the literature (McGinn et al., 2016) was carried out through the Scopus, Web of Science and PubMed databases for search of abstracts and contents. We followed the procedures related to the literature study, such as use of general keywords, identification of a research topic (Ethnic minorities user perspective in mental health services) and concept maps definition (intersection circles between different themes and processes), in the qualitative research field.

The criteria adopted were: (a) European context (Services), (b) Papers written in English, (c) We were exclusively interested in the perspective of users belonging to ethnic minorities who described or reported experiences of discrimination and stigma perceived directly when accessing mental health services, (d) quantitative and qualitative studies. We used the following main search string: “Effects OR consequences OR responses/AND from the perspective of mental health services users belonging to ethnic minorities,” with different combinations. Different synonyms are also used, as: perception user, view, feelings, minorances, users perspective, mental health services, ethnology, inequalities, migrant groups, Brain Services, Psychology/Psychiatry Services.

Concerning exclusion criteria, we have excluded studies (a) that do not explicitly quote the users’ perspectives, (b) that do not regard Mental Health Services, (c) that do not concern European Locations.

From an initial research, we obtained 3794 abstracts, many of which were immediately excluded as they did not focus on the psychology area or ethnic minorities, then many others were excluded as they did not focus on the ethnic users perspective or on the ethnic inequalities/barriers of access to mental health services. Of these, 618 studies were considered relevant, although 584 were excluded because they were about mental health services staff’s perspective or not including the Europe context or the English language. The significant reduction in selected articles stems from the fact that we initially chose broad keywords that made it possible to obtain a large number of articles. One of the requirements was a focus on services, and within services we chose articles that highlighted either keywords or references to keywords used by ethnic minority users. This focus is not more frequent in the literature.

**FIGURE 1:**
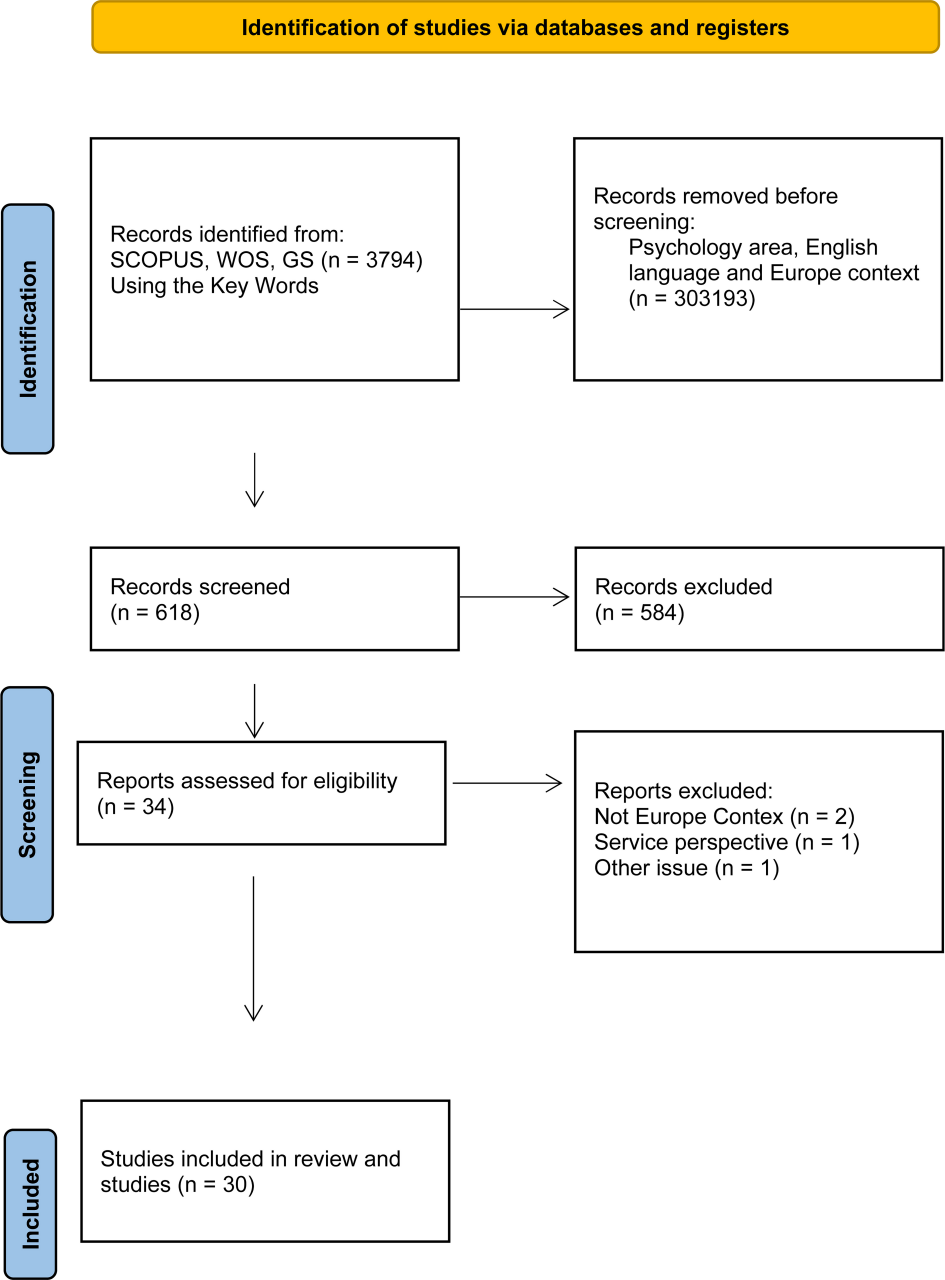
Flow diagram illustrating the processes of literature searches and screening.

Actually, only studies concerning individuals from diverse ethnic backgrounds who seek help in mental health services in Europe were included. Eventually, just 30 studies were considered (Table 1). The research period was 2003–2025 (see Figure 1).

**TABLE 1:** Study selection and characteristics.

	References	Country	Objective(s)	Sex assigned at birth	Average age	Country of origin	Type of study
1	Ali et al., 2013	United Kingdom	To examine the extent to which patients with intellectual disability and their carers experience discrimination or other barriers in accessing health services, and whether health care experiences have improved over the last decade years	Both	23–57 years	Asian Indian; Asian Pakistani origin	Qualitative
2	Bansal et al., 2022	United Kingdom	To provide a new conceptual understanding of how ethnic inequalities are created and sustained; this is essential to develop effective interventions	Both	Not indicated	Unknown BME	Mixed methods
3	Bassey and Zaka, 2024	United Kingdom	To uncover barriers to MHS utilization and proffer evidence-based recommendations toward addressing the mental health needs of African immigrants residing in the UK	Both	Not indicated	African/Afro-Caribbean origin, Somali refugees, and ethnic minorities of Black/British descent	Mixed methods
4	Bonell et al., 2012	United Kingdom	To establish whether there are any differences in the experiences of people with ID and mental health problems from two ethnic com munities in South London	Both	36–51 years	Black British, Black African or Black Caribbean	Mixed methods
5	Chui et al., 2021	United Kingdom	To (i) identify inequalities in referral source by age, ethnicity, migration status, and gender, (ii) examine differences in referral destination by age, ethnicity, migration status, and gender, and (iii) examine associations between referral source and referral destination	Both	12–29 years	Black Caribbean, Black African, Asian, Mixed	Quantitative
6	Abebe et al., 2017	Norway	To examine the use of specialist mental healthcare services among ethnic Norwegians and specific immigrants groups	Both	0–59 years	Sweden, Poland, Bosnia-Herzegovina, Russia, Somalia, Turkey, Sri Lanka, Iraq, Iran, Pakistan, Vietnam	Quantitative
7	Ekezie et al., 2023	United Kingdom	To explore the health and social care experiences of ethnic minorities and other minoritized populations, their research interests and appropriate research practices.	Both	25–75 years	African Caribbean, Eastern European, Somali and South Asian communities	Qualitative
8	Czapka and Sagbakken, 2016	Norway	To identify the main barriers and facilitators experienced by post-accession Polish migrants in accessing and utilizing health care services in Norway	Both	20–60 years	Polish migrants	Qualitative
9	Fazel et al., 2005	United Kingdom	To search for psychiatric surveys that were based on interviews of unselected refugee populations and that included current diagnoses of post-traumatic stress disorder, major depression, psychotic illnesses, or generalized anxiety disorder	Both	Not indicated	Southeast Asia Other	Mixed Methods
10	Halsall et al., 2025	United Kingdom	To explore perinatal mental health occupational therapists’ perceptions of the barriers and enablers to an inclusive service provision for ethnic minority mothers.	Women	Not indicated	Mixed background	Qualitative
11	Hardy et al., 2025	United Kingdom	To explore the perspectives of racially minoritized students on help-seeking for mental health problems by asking the question “What are the attitudes toward seeking help for mental health problems among racially minoritized students?” A secondary aim of the review was to explore how universities and mental health services can support help-seeking for mental health among racially minoritized students.	Both	18–28 years	Afro-Caribbean, Black British, South Asian-British, British Muslims from Arab, Asian, African heritage, South Korea, USA, Arab, China, India, Vietnam, Filipina, Malaysian, Saudi Arabian, Indonesian, Iranian	Qualitative
12	Harwood et al., 2021	United Kingdom	To provide equitable access to therapy for common mental disorders	Both	At least 16 years	Black African, Asian, Black Caribbean, Black Other, White Other, Mixed	Quantitative
13	Bhui et al., 2003	United Kingdom	To identify ethnic variations in pathways to specialist mental health care, continuity of contact, voluntary and compulsory psychiatric in-patient admissions; to assess the methodological strength of the findings	Both	Not indicated	Black Caribbean, Asian, Black African, Indian, African Caribbean, Black other, Other European, Jamaica, Barbados, Irish, Unknown	Quantitative
14	Knifton, 2012	United Kingdom	To promote positive mental health for all, prevent common disorders, and enhance the quality of life of people with mental illness	Both	Not indicated	Pakistani, Indian and Chinese heritage	Qualitative
15	de la Cruz et al., 2015	United Kingdom	To compare the clinical characteristics of a sample of White vs. non-White children and adolescents with OCD treated at a national specialist clinic in the UK; to test whether the outcomes of a multimodal, evidence-based treatment for OCD were comparable in both groups	Both	Children and adolescents	Identified themselves as belonging to a White ethnic background (including 135 British, 28 English, and 6 “other White” individuals) and 35 to a non-White ethnic background (including 14 Mixed, 8 Black, 6 Asian, and 7 “other ethnic minority” individuals).	Quantitative
16	de la Cruz et al., 2015	United Kingdom	To explore whether ethnic minorities with OCD are underrepresented in secondary and tertiary mental health services in the South London and Maudsley (SLaM) NHS Foundation Trust	Both	Not indicated	(a) White (including British, Irish and any other White background groups); (b) Mixed/multiple ethnic groups (including White and Black African, White and Asian, and any other mixed background groups); (c) Asian/Asian British (including Indian, Pakistani, Bangladeshi, Chinese and any other Asian background groups); (d) Black/African/Caribbean/Black British (including African, Caribbean and any other Black background groups); and (e) any other ethnic group	Quantitative
17	Fernández de la Cruz et al., 2016a,b	United Kingdom	To shed new light on the reasons for these inequalities.	Both	36.9	Black African, Black Caribbean, Indian	Quantitative
18	Lynch et al., 2018	United Kingdom	To explore barriers to professional help seeking for mental health problems among young men (18–24 years) and to explore solutions proposed by them that are relevant to their lived realities	Men	18–24 years	Northern Irish, Irish, Scottish, Greek American, Chinese	Qualitative
19	Aichberger et al., 2015	German	To assess the extent of the relationship between perceived ethnic discrimination and psychological distress among women of Turkish origin living in Berlin, Germany, and to explore whether this association is moderated by acculturation strategies while controlling for other known predictors of distress in migrant populations	Women	18–75 years	Turkish origin	Quantitative
20	McMullen et al., 2024	United Kingdom	To explore the potential role of culturally relevant and adapted social prescribing in assisting Pakistani carers and identify the cultural and religious influences and barriers on carer health behaviors	Both	At least 18 years	Pakistani	Qualitative
21	Priebe et al., 2012	Europe	To describe the characteristics of services providing mental health care for people with mental disorders from socially marginalized groups in European capitals	Both	Not indicated	Not indicated	Mixed methods
22	Priebe et al., 2013	Europe	To identify components of good practice in the provision of mental health care across six groups that are widely considered as socially marginalized	Both	Not indicated	Not indicated	Qualitative
23	Henderson et al., 2015	United Kingdom	To explore the role of psychiatric admission, diagnosis and reported unfair treatment in the relationship between ethnicity and mistrust of mental health services.	Both	At least 18 years	Self-defined Black, White or Mixed (either Black and/or White mixed) ethnicity	Quantitative
24	Ayub and Macaulay, 2023	United Kingdom	To explore the perceptions of the British Pakistani Muslim community toward mental health and barriers toward seeking treatment	4 women 3 men	20–40 years	Pakistani origin	Qualitative
25	Robertson et al., 2019	United Kingdom	To summarize what is known about the health status of those with intellectual disabilities from minority ethnic communities in order to document potential health inequalities and identify gaps in knowledge; and to provide a narrative synthesis of research relating to the physical or mental health care of people with intellectual disability from minority ethnic communities in order to provide potential directions for future research, policy and practice	Both	5–93 years	Asian Pakistani, Asian Indian, Black Caribbean and African, South Asian, African heritage, Bangladeshi, Afro-Caribbean, Middle East/Arab, Mixed Ethnicity, East African Asian, Indian,	Quantitative
26	Nwokoroku et al., 2022	United Kingdom	To synthesize and summarize evidence on the role of culture in MH service utilization among ethnic minorities in the UK	Both	At least 18 years	Black Caribbean; South Asia, Black Africa and the Black Caribbean; BME community; Black African and African Caribbean;	Quantitative
27	Kolvenbach et al., 2018	United Kingdom	To identify and compare barriers that parents from different ethnic groups face when accessing specialist services for obsessive–compulsive disorder (OCD) for their children	Both	Parents mean age of 45.8 years. Children’s mean age 15.35	Black Africans, Malaysian, Indian, Black Caribbean, Iranian, and Pakistani	Qualitative
28	Alam et al., 2024	United Kingdom	To understand the barriers to accessing formal mental health support for racially-minoritized people within the UK	Both	18–65 years	Black Caribbean, Pakistani, Somali, Indian, Bangladeshi and Chinese. Other studies used broader terms to describe participants such as African, South Asian, Latin American or Mixed Heritage	Qualitative
29	Fassaert et al., 2016	Netherlands	To test the hypothesis that ethnic minority status of patients is associated with specific psychotic disorder treatment characteristics	Both	18–65 years	Ethnic Dutch, Antillean, Surinamese, Moroccan, Turkish, Other non-western, Other western, Ethnicity unknown	Quantitative
30	Kieseppä et al., 2020	Finland	To compare the intensity of psychiatric care, as an indicator of treatment adequacy, between natives and immigrants living in Finland	Both	The categories were: (1) 15–29, (2) 30–44, (3) 45–59, and (4) 60 years or more.	(1) EU/European Free Trade Association (EFTA), North America, and Australia, (2) Eastern Europe (including Russia and the former Soviet Union), (3) the Middle East and Northern Africa, (4) Sub-Saharan Africa, and (5) Asia	Quantitative

### 2.3 Methodological quality assessment

We carried out a qualitative analysis of the documents covered by the criteria (Creswell, 2013). The authors of this work supervised the selection process, which occurred in three distinct stages. First, articles were analyzed according to the inclusion criteria; second, papers were evaluated in terms of their titles and abstract; and third, they were directly acquired. These final articles were studied and analyzed through a rigorous content analysis. Given the complexity of the topic, we divided the general theme into sub-concepts, called intersection areas, and entered them in an Excel spreadsheet. Several columns were created to divide the fundamental data according to the country of study, prevalent population, ethnic minority population, age of the persons involved, type of clinical service taken into consideration, type of article considered, methodology used by the authors, key findings, clinical implications, discussion, and limitations of these studies. As with any other analytical method in qualitative research, documents analysis required data to be examined and interpreted to obtain sense and understanding. This process also aimed to develop empirical knowledge (Rapley, 2011; Corbin and Strauss, 2008). The entire text selection and analysis process was initially done individually and then together in a second phase, during which we discussed the differences. Based on the type of our work, the critical appraisal tool used in this research is the CASP (Critical Appraisal Skills Programme) Systematic Review checklist (2023). This checklist is based on several criteria including: Focused Question, Right Papers, Quality Assessed, Reasonable Combination, Overall Results, Precision, Local Application, Outcomes Considered, Benefits Worth Costs. Using the criteria for assessing methodological quality and risk of bias based on the CASP list the results are categorized as high quality, moderate quality, and low methodological quality. Our work achieved 66.66% high quality, 26.66% moderate quality, and 6.66% low quality. These values are therefore considered to be of significant quality overall.

## 3 Findings

The results emerged from a meticulous review and analysis of the selected articles. The articles were studied to draw up a table containing the results from each. The two main results were selected based on the frequency with which they were repeated in all the articles. Therefore, the two macro categories emerged as the most frequently cited and reported by users belonging to ethnic minorities. The results were broadly subdivided into the following macro-categories: (1) difficulties to access to services and (2) the perception of unequal or inappropriate treatment.

In the first category, we have grouped together the processes that lead to non-use of services, i.e., barriers that prevent access to services. In the second category, we have grouped together the theories and experiences of minorities with regard to the actual treatment offered by the services.

### 3.1 Difficulties to access to services

#### 3.1.1 Feeling discriminated against

The perception of discrimination, defined as the sensation of being treated unequally compared to the majority population, is particularly relevant in mental health services. Williams and Mohammed (2009) describes it as a “psychosocial stressor,” impacting individuals’ well-being and influencing access to services.

Ethnic minorities who come into contact with mental health services frequently report situations of distrust and skepticism, stemming from perceptions of discrimination received from medical staff and the services themselves. This phenomenon is particularly evident for individuals of Black or mixed ethnic backgrounds, as highlighted by Czapka and Sagbakken (2016). Supporting this observation, Henderson et al. (2015) reports significant differences in hospitalization proportions among ethnic groups, with higher rates for people of Black or mixed backgrounds compared to the White population. Research further demonstrates that, despite considerable differences among minority groups, ethnic background is generally associated with shorter waiting times, but also a greater number of police referrals, emergency contacts, clinical facility admissions, and compulsory hospitalizations, with a shorter treatment duration. Paradoxically, the shorter waiting times for patients with minority ethnic backgrounds in this study are indicative of less favorable pathways. This suggests that faster access to services does not always translate into higher quality or more appropriate care for these groups (Chui et al., 2021). Ethnic groups are more likely than White individuals to be directed to treatment in inpatient services rather than outpatient services (Oduola et al., 2021). Some of the articles included in this work examined inequalities in both the source of referral and the destination of referral, using data from electronic mental health care records. It has also been found that migrants are more often admitted to compulsory care because they are considered a danger to others (Harwood et al., 2021). Further research has delved into the experiences of users from ethnic minorities, revealing a concerning picture. These users reported often being ignored, rejected, judged, receiving inadequate reception, and being treated rudely and differently compared to White users (Ekezie et al., 2023; Lynch et al., 2018). Such negative experiences were primarily attributed to their language and skin color. The lack of mental health awareness and poor understanding of symptoms are also influenced by racial/ethnic discrimination, further exacerbating the situation for these minority groups (Ekezie et al., 2023).

#### 3.1.2 Fear of stigma and labeling

Ethnic beliefs significantly influence how ethnic minorities perceive and experience discrimination in mental health settings (Lynch et al., 2018).

Stigma can be both internal (self-criticism) and external (medical critical voice), influencing help-seeking behavior and perceptions of diversity and discrimination (Memon et al., 2016; Ekezie et al., 2023). The attribution of psychiatric diagnoses may be perceived as a form of labeling and inescapable stigmatization, leading many individuals from ethnic minorities to resist or reject such labels. This resistance, whilst potentially a coping strategy, may also impede access to necessary care (Edge and MacKian, 2010).

Ethnic perceptions of mental health can profoundly influence the experience of stigma. In some communities, mental health problems may be viewed as a negative reflection not only on the individual but on the entire family or ethnic group, thus amplifying the impact of stigma (Shefer et al., 2013). Experiences of racial discrimination can intersect with mental health-related stigma, creating multiple and complex barriers to accessing services for ethnic minorities (Rabiee and Smith, 2014). Stigmatization can be defined as a form of discrimination that occurs in circumstances of power imbalance, such as in the relationship between service provider and user (Iudici et al., 2021; Mantovani et al., 2017; Turchi et al., 2022a,b). This definition highlights how stigma can be intrinsic to the very structure of mental health services, manifesting through unbalanced power dynamics.

#### 3.1.3 Shame and family judgment as a barrier

Shame and family judgment are significant barriers for ethnic minority users. Fear of being considered different and bringing shame to the family limits opportunities within the ethnic community and associates negative connotations with therapy (Hardy et al., 2025).

Mental health-related shame can be amplified by ethnic factors, experiences of racism and discrimination, and the perception of being “different” or “inferior” in the context of the dominant society (Ekezie et al., 2023). These experiences often lead to situations of exclusion and the sensation of being judged (McMullen et al., 2024).

Ethnic minorities face unique barriers in accessing mental health services, which extend beyond the individuals requiring care to their families and communities (Alam, 2023; Alam et al., 2024). A primary concern for these families is the fear of community judgment, reflecting the deep interconnection between the individual and their community in minority cultures (Kolvenbach et al., 2018). Stigma and judges experienced within communities and families themselves add an additional layer of complexity. These factors can lead parents to feel shame about their child’s mental illness, creating a cycle of silence and denial that further hinders access to necessary care (Kolvenbach et al., 2018). The embarrassing and sometimes taboo nature of mental disorder symptoms may be associated with higher levels of shame and stigma in some ethnic groups, making it more difficult for individuals with mental disorders to seek help (Shefer et al., 2013; Memon et al., 2016). Addressing these challenges requires a long-term commitment to change not only clinical practices but also broader social perceptions of mental health in diverse ethnic communities. A holistic and essential approach is necessary to overcome barriers related to shame and judgment in mental health care for ethnic minorities.

#### 3.1.4 Minimization of need and alternative access to services

The underrepresentation of ethnic minority patients in clinical mental health services is a complex issue leading to unmet treatment needs (de la Cruz et al., 2015; Goodman et al., 2008). Reasons for this remain largely unknown and cannot be fully explained by differences in prevalence rates. One such factor could be the perception of mental disorders. Ethnic minorities might perceive mental disorders as less disabling, leading to reduced help-seeking behavior. However, evidence supporting this hypothesis requires further corroboration (de la Cruz et al., 2015).

Another salient aspect concerns alternative sources of help. Individuals from ethnic minorities might be more inclined to seek assistance from resources other than traditional mental health services, such as elderly relatives, religious institutions, or the community (de la Cruz et al., 2015). This tendency could contribute to the underutilization of conventional mental health services. Moreover, access pathways to services could vary significantly between ethnic minorities and the majority population (Williams et al., 2012a). These variations in the ways different communities come into contact with mental health services could influence the utilization and efficacy of interventions. Understanding these dynamics is fundamental for developing targeted strategies aimed at improving access to and utilization of mental health services by ethnic minorities, taking into account the specific challenges and ethnic nuances of these communities.

### 3.2 The perception of unequal or inappropriate treatment

#### 3.2.1 Disparities, mistrust, and perceived injustice

Ethnic minorities express significant mistrust in the mental health system, particularly in relation to their ethnic group compared to the white majority. This mistrust, defined by Hoy and Tschannen-Moran (1999) as the willingness to be vulnerable based on the belief in another’s benevolence, reliability, competence, honesty, and openness, is crucial in mental health contexts where patient vulnerability is essential for effective treatment. The mistrust stems from perceived unfair treatment by mental health services and staff (Czapka and Sagbakken, 2016). Armstrong et al. (2013) suggest it may result from greater exposure to negative experiences with mental health services. Rose et al. (2011) highlight that “unfair treatment” often relates to feeling unheard, particularly relevant for ethnic minorities who may feel marginalized within the healthcare system. This sentiment is captured by a participant’s testimony: “You’re supposed to have a bit of trust in the system, which I guess we don’t have now because the system has failed us so badly” (Male, 47 years, African; Czapka and Sagbakken, 2016). This quote reflects the disillusionment many ethnic minorities harbor toward the healthcare system. Addressing these disparities and rebuilding trust requires a multifaceted approach that acknowledges and incorporates the unique experiences and perspectives of ethnic minorities in the context of mental health (Bassey and Zaka, 2024).

#### 3.2.2 Ethnocentric approach and cultural insensitivity

The ethnocentric approach in mental health services presents a significant barrier for ethnic minority users. Harwood et al. (2021) note that general practitioners may lack qualification to detect mental disorders on an ethnic basis or hesitate to approach reluctant patients presenting disorders for the first time. The rigidity of certain treatments, often stemming from the biomedical model, can alienate users unable to grasp their significance, particularly those from minority cultures with specific ethnic needs (Lu et al., 2021). Users express frustration with the inadequacy of treatment in addressing linguistic, cultural, social, and ethnic differences or providing culturally appropriate assistance.

This frustration encompasses the lack of cultural sensitivity and inclusion of alternative conceptualizations of mental distress that deviate from the “pure” or “medical” model. Moreover, the perception that racial and cultural issues do not adapt to the biomedical model seems to be widespread. There appears to be a lack of understanding of culturally different interpretations of illness and healing. Ethnocentric and biomedical approaches are often perceived as stigmatizing and inadequate to represent the problems experienced by users. Even psychiatrists often view this from the perspective of “white doctors” (Bansal et al., 2022).

Explanatory models (i.e., the patient’s understanding of illness) of patients from ethnic minorities may be different and require further investigation (Williams et al., 2012b). Some ritualistic behaviors could be erroneously interpreted as part of religious rituals and thus not recognized as clinical problems (Memon et al., 2016).

The importance of considering cultural differences is emphasized, especially for people from diverse backgrounds who might encounter additional barriers during the transition to mental health services (Oduola et al., 2021). This underscores the necessity for a more inclusive and culturally sensitive approach that accounts for the diverse perspectives and experiences of ethnic minorities in mental health contexts.

#### 3.2.3 Perception of neglect and unmet expectations

Users from ethnic minority backgrounds often report experiences of neglect in their interactions with health services, manifesting in various forms and contexts. Experiences of migrant women feeling neglected by doctors solely due to their migrant status have been documented in several studies. For instance, two young women of Albanian origin described their treatment perceived as overtly discriminatory regarding breast examination guidance. One felt discriminated against for the importance of breast self-examination, which was not emphasized, whilst another interpreted the nurse’s “insensitive” behavior as discriminatory compared to non-migrant mothers (Abebe et al., 2017).

Many migrants have reported concerns about treatment practices and medication prescriptions. Prescriptions for mild antidepressants or recommendations for rest have often been interpreted as signs of discrimination and have led to a mistrust of doctors. Some migrants equate treatment solely by a doctor’s prescription to a form of discrimination, particularly for antibiotics. Indeed, in their countries of origin, many were accustomed to various treatment methods, including more or less frequent use of antibiotics (Madden et al., 2017). These negative experiences create a significant distance between ethnic minorities and medical assistance services, rendering many members of these communities less inclined to seek care or follow recommended therapies. Addressing these perceptions and improving ethnic communication in the healthcare context is fundamental to ensuring equitable access and quality care for all population groups (Robertson et al., 2019). Further aspects of this perception of neglect have been highlighted in various studies. There is a widespread belief among migrant patients that they are less likely to receive offers of verbal therapy and more likely to receive pharmacological therapy compared to white patients. This is accompanied by situations where verbal therapy, despite being desired and explicitly requested, is not offered. Moreover, there has been a reported perception of an increase in violence by staff toward patients of color. Particularly concerning is the perception, shared by some service providers, that within the healthcare system there is a conviction that people of color cannot be effectively treated with verbal therapy (Bansal et al., 2022). These experiences and perceptions underscore the need for a more culturally sensitive and inclusive approach in health services, which takes into account the diverse expectations and backgrounds of patients from ethnic minority communities.

## 4 Discussion

As a result of the categories and the presentation of the results, it was possible to identify a diversity of criticalities reported by minority users. The issue of discrimination emerges clearly from the very first moment when people from ethnic minorities contact mental health services. From an interactionist perspective, we focused our attention on the voices of ethnic users, who report distrust regardless of admission (Memon et al., 2016).

The lack of trust manifests despite users achieving treatment at certain services for various reasons, such as increased experience of unfair treatment, distrust of health care based on personal experiences of racial discrimination, or distrust based on hearing about others’ bad experiences with mental health services. Unfair treatment as a latent element most often referred to interactive issues such as not feeling heard. This influences a significant under-representation of minority patients in clinical services (Bhui et al., 2003).

Recent studies have highlighted the need for a comprehensive approach to address these issues. Evangelidou et al. (2023) points out the need for a public health paradigm shift that actively engages both vulnerable migrant refugees (VMR) and professionals in decision-making processes to enhance access and promote health strategies. This approach aligns with recommendations for both “upstream” measures (such as data collection and governance) and “downstream” measures (like improving access to health services and responsiveness to migrants’ needs). A “Road Map” has been proposed that emphasizes both “upstream” measures (data collection, governance, intersectoral action on social determinants of health) and “downstream” measures (access to health services, responsiveness to migrants’ needs, attention to vulnerable groups). Importantly, migrant status can have both direct and indirect effects on health, often mediated by socioeconomic position (SEP). Nordström et al. (2020) emphasizes the urgent need to implement solutions to migrants’ health inequalities, addressing socioeconomic inequities and those related to migration and ethnicity in tandem, rather than as separate issues.

One of the assumptions of the interactionist approach reports that constructive and interpretative acts of events depend on the relationships in place, the context and the related roles, rules and value judgments (Burr, 2015; Turchi et al., 2023; Hacking, 1999). This perspective emphasizes the importance of social interactions and ethnic context in shaping individuals’ perceptions and experiences of mental health services (Kirmayer et al., 2018; Turchi et al., 2019).

Evagora-Campbell et al. (2022) notes a paucity of research addressing the structural determinants of health inequities in labor migrants, despite the rise in international migration. This gap in research underscores the need for the academic community to pay greater attention to structural determinants of health, which often requires cross-disciplinary and cross-sectoral collaboration.

The construction of relationships with people and the healthcare context can become critical to such an extent that not only do they fail to establish positive relationships with the appropriate personnel, but also lead to users not feeling listened to and understood regarding their difficulties and discomforts. It seems of paramount importance to understand the reasons for inequalities in the way users are received and treated to resolve them. Ethnic minorities are often faced with substantial unmet treatment needs (Alegría et al., 2017). Ethnic Minorities who eventually access services may not be fully representative of their minority group as a whole. For example, patients who access services may be those who accept the “western” view of how to overcome mental health problems, whereas people who do not access treatment may have a different view of treatment and may respond differently to the mental health resources offered (Kirmayer and Ryder, 2016).

There is a need for public health professionals to work to reduce inequalities in access to services. The interactionist approach brings back the pivotal point of understanding the other’s world, of remaining as close as possible to the text produced by the patients as in the configurations or interpretations of reality there are not things or objects, but entities that acquire meaning through the use of language. Attention to detail in planning, the use of relevant incentives and targeted community outreach efforts seem to be helpful in engaging ethnic minorities in clinical settings. Ethnic modifications to empirically supported treatments, such as cultural matching with the therapist, the use of a culturally sensitive therapist, the use of culturally appropriate examples, or the use of culturally rooted strategies have been suggested as relevant strategies that may contribute to improved help-seeking and treatment compliance (Benish et al., 2011).

Possible explanations for the under-representation of ethnic minorities in mental health services include the perception of some symptoms or disorders as being of little harm, which does not prompt users to seek help despite their symptoms. Alternatively, the embarrassing and sometimes taboo nature of symptoms may be associated with higher levels of shame and stigma in some ethnic groups due to the way they perceive psychiatric services, making it more difficult for people to actually seek help.

It is also possible that the explanatory models (i.e., the patient’s understanding of the illness) of minority patients are different and need to be further investigated (Kleinman and Benson, 2006). Some ritualistic behaviors may not be considered as clinical problems. Additionally, individuals from ethnic minorities may be more likely to seek help from resources other than mental health services (e.g., elderly relatives, religious institutions or communities) or the way in which they come into contact with services may vary.

The interactionist approach allows us to broaden the possibilities of understanding while avoiding value judgments specific to European culture and the social context in which we are embedded (Burr, 2015). Roles, rules and value judgments are personal and interpersonal, and enable the decoding of meanings, acts of construction and interpretation of reality (Berger and Luckmann, 1966; Gergen and Gergen, 2012). Such acts also lead the attributions of meaning to be socially constructed.

Ethnic minorities are people who may attribute different meanings to symptoms, disorders or contacts with health professionals than the prevailing population’s way of constructing reality (Kirmayer et al., 2018). Experiences and traditions are different, the way they come into contact with mental health services is different, and to date, this cannot be ignored.

Users report a significant sense of judgment, stigma and discrimination (Memon et al., 2016). The debilitating effects of discrimination go beyond the influence of known risk factors for psychological distress, such as unemployment, being single, having a limited residence permit and even in the presence of personality structures that may increase vulnerability for stress responses and mental disorders (Pascoe and Smart Richman, 2009).

Ethnic minorities tend to be confronted with individuals belonging to their culture, their traditions, and in short, their family or peers. Often, family members report negative experiences and note critical issues when approaching health care facilities. This generates a mechanism of mistrust, lack of trust, and powerlessness that is passed on from generation to generation (Alegría et al., 2016).

Ethnic minority families in Europe are difficult to reach for the prevention and treatment of various psychological disorders (Reardon et al., 2014). It is assumed that one reason for the low engagement of some parents of children in mental health services is their tendency to perceive, in comparison to ethnic majority families, the externalizing and internalizing behavior of children as less problematic.

Religious or traditional values such as collectivism and conformity, rather than individualism and authenticity, may influence the way ethnic minorities perceive atypical behavior or symptoms of children or family members, and the extent to which they feel personally affected by this behavior (Kirmayer and Ryder, 2016). This ethnic difference leads them to be more inclined to focus on adapting to the needs of their relatives, rather than feeling personally oppressed.

Families from ethnic groups who fear stigmatization may be more reluctant to acknowledge that their family members may have behavioral difficulties. Most of the ethnic minorities families analyzed in the studies lived in deprived neighborhoods and had a lower socio-economic status. Socio-economic status often predicted treatment intensity: subjects who were not employed were more likely to receive low-intensity treatment (Delgadillo et al., 2016).

Low income has been linked to a greater need for mental health treatment. Very often, difficulties with communication and language skills, a perceived sense of discrimination, and socio-economic difficulties lead people from ethnic minorities to perceive and report inadequate treatment as a consequence (Sentell et al., 2007).

The fact that ethnic minorities seem to benefit from evidence-based treatments as much as their white counterparts, yet are under-represented in clinical services (de la Cruz et al., 2015), denotes that ethnic minorities face substantial unmet treatment needs. This may further contribute to the socio-economic hardships that some ethnic minority groups already face (Alegría et al., 2017).

In conclusion, addressing the inequalities in access and treatment of mental health services for ethnic minorities requires a multidimensional approach. This approach should take into account cultural differences, socio-economic barriers, and the need to build trust through culturally sensitive practices. By understanding and addressing these complex factors, we can work toward more equitable and effective mental health services for all members of society.

## 5 Conclusion

On the basis of the above considerations, it is possible to outline a few points on which future studies should focus, or at least on the changes that scientific research urges in order to achieve an adequate movement to the needs of ethnic minorities in the field of mental health. The cross-sectional designs presented in the research allow us to estimate the effects of historical events such as psychiatric hospitalization on current views such as distrust, but critically we cannot determine the impact of distrust and unfair treatment on subsequent clinical and service utilization outcomes. First and foremost, this represents important initial future work. In fact, there is a strong need for a renewal of resources and tools to design and test interventions aimed at reducing ethnic disparities in the use of mental health services, also considering the unfair treatment patterns within the services reported by the users themselves (Memon et al., 2016).

These operations would in any case lead to an increase in trust in the services and a word-of-mouth of positive experiences passed on from generation to ethnic generation. It should not be underestimated that the anticipation of such issues would guarantee a better quality of service provision, which would better meet the real and felt needs of users, and which would in turn guarantee an improvement in therapeutic collaboration between professionals and patients. In fact, experiences of care and ease of access to care would be a protective element against psychological distress (Alegría et al., 2016).

In the studies exposed through this work, it was found that knowing that support was available and how it could be structured, helped individuals to seek help and access services sooner. Positive and even transformative experiences were attributed to the feeling of being understood, listened to and validated. This can be verified through the ways of curiosity, kindness, flexibility and trust-building that mental health professionals adopted toward ethnic minority patients (Kirmayer et al., 2018).

The awareness and realization that there are inequalities in the way ethnic minorities are treated compared to the prevailing populations highlights that more needs to be done to understand the causes of the known ethnic disparities in the diversification of mental health services, and to implement strategies based on the goal of reducing and eliminating these inequalities (Bhui et al., 2015). The implementation of collaborative mental health initiatives between professional health services and voluntary, community and social enterprise organizations can improve understanding and dispel myths about mental health. It can also increase access to culturally competent professional mental health services, improving satisfaction with mental health care received among black populations (Halvorsrud et al., 2018; Bassey and Zaka, 2024).

When undertaking research that examines mental health by ethnic groups, tracking mental health prevalence and treatment outcomes by precise ethnic groups will greatly assist in monitoring trends in this area and ensuring that progress is made toward a more equitable experience for all (Jongsma et al., 2019). So far, it appears that physicians and politicians have had little information regarding the disparities in diagnosis and treatment of minority groups. For this reason, efforts are needed to ensure sensitivity to different cultural values regarding mental health and to encourage recognition of the symptoms and help-seeking behavior of these populations, taking into account the fact that different cultural values take on different entities and guises than the prevailing culture of a given country (Kirmayer and Ryder, 2016).

It is of paramount importance to understand whether there are differences in mental health beliefs and treatment preferences between different ethnic groups, as strategies that work for the Italian population, may not be functional and effective for minority populations. Politicians and doctors should be well aware of this. Differences exist and partly explain the gap in mental health care and treatment. However, if differences in illness perception and help-seeking behavior are minimal or do not exist, then it becomes crucial to search for, understand and eventually eliminate the barriers to more equitable care (Rathod et al., 2018). Further studies should focus on the reasons for these ethnic inequalities in order to ensure good quality mental health care for all ethnic groups.

Overall, perceived ethnic discrimination was found to negatively influence psychological distress even controlling for known social factors, migration-related factors and personality traits associated with distress (Pascoe and Smart Richman, 2009). The results suggest several important directions for the development of future practices, policies and research. High quality services are the result of community involvement, understanding the needs and wishes of patient or client populations and adapting to their needs as far as possible in each service encounter (Kirmayer et al., 2018). Similarly, policies should be congruent with the needs of the people involved and should be informed both by impacts on individuals and by a macroscopic understanding of local communities as a whole.

The moderating role of acculturation could be explored to a greater extent to gain a more complete understanding of the role of adaptation in health and mental health (Berry, 2019). Variations within groups also appear to support the individualization of services. Exploring what interventions are effective, for whom and what kind of outcomes they influence continues to be important in health and mental health service provision and is critical to establishing cultural competence and promoting health and mental health in our diverse and ethnic societies (Benish et al., 2011; Rathod et al., 2018).

## 6 Limitations

First, this review is limited in its concerns over the scarcity of specific studies involving patients from different ethnic backgrounds. It focuses on research conducted in the Western academic world; when discussing ethnic minorities groups or migrants from unfamiliar areas, there is, thus, the risk of taking many concepts for granted. It is important to underline that most of the studies we examined were conducted in some places in the UE context. This factor may have guided the results and analyses, and therefore, one must tread with caution in generalizing the results, also because these results concern some Services and not a specific European institution. Furthermore, there was considerable variability among the studies included, such as the number of participants, survey methods and evaluation of the interventions. Future studies should aim to investigate the topic under review in different and more specific contexts, e.g., the Italian context.

Further research is needed on young people’s journeys from adolescent to adult services and how inequalities in referral source may impact treatment pathways and patient outcomes. That is a huge issue for people with different background. It is crucial to understand the reasons for these inequalities and resolve them. These may be related to possible referral barriers, obstacles in accurate diagnosis post-referral to mental health services, or differences in beliefs about mental health care and treatment preferences across different ethnic minorities groups.
